# Assessment of the molecular identification algorithm and its impact on antifungal susceptibilities against clinical *Fusarium* isolates: a multicentre study in Taiwan, 2011–2023

**DOI:** 10.1093/jacamr/dlag022

**Published:** 2026-02-26

**Authors:** Pao-Yu Chen, Chi-Jung Wu, Un-In Wu, Wang-Da Liu, Yee-Chun Chen

**Affiliations:** Division of Infectious Diseases, Department of Internal Medicine, National Taiwan University Hospital, Taipei, Taiwan; Division of Infectious Diseases, Department of Internal Medicine, National Cheng Kung University Hospital, College of Medicine, National Cheng Kung University, Tainan, Taiwan; National Institute of Infectious Diseases and Vaccinology, National Health Research Institutes, Miaoli, Taiwan; Division of Infectious Diseases, Department of Internal Medicine, National Taiwan University Hospital, Taipei, Taiwan; Department of Medicine, National Taiwan University Cancer Center, Taipei, Taiwan; Division of Infectious Diseases, Department of Internal Medicine, National Taiwan University Hospital, Taipei, Taiwan; National Institute of Infectious Diseases and Vaccinology, National Health Research Institutes, Miaoli, Taiwan; School of Medicine, National Taiwan University College of Medicine, Taipei, Taiwan

## Abstract

**Objectives:**

Invasive fusariosis is rising and poses challenges due to species complexity and antifungal resistance. *In vitro* susceptibilities of new antifungals against *Fusarium* isolates are seldomly evaluated in Asia. This study aimed to evaluate a two-step molecular identification algorithm and to perform *in vitro* antifungal susceptibility with correlation of species and susceptibility patterns.

**Methods:**

*Fusarium* clinical isolates collected at three hospitals in Taiwan (2011–2023) were identified to species level using sequential *ITS* and *TEF1α* sequencing (step I), followed by *RBP2* sequencing (step II) for inconclusive isolates. Minimum effective/inhibitory concentrations (MECs/MICs) of manogepix, olorofim, amphotericin B and voriconazole were determined by EUCAST method (E.Def 9.4).

**Results:**

Of 103 isolates (37 blood and 66 cornea isolates) evaluated, the two-step algorithm achieved >90% to species level. *Fusarium solani* species complex (*FS*SC) was predominant, especially in blood isolates (86.5% versus 65.2% in cornea isolates; *P* = 0.02). The rest 28 isolates belonged to 12 species within six species complexes (SCs). Manogepix exhibited potent activity against all isolates (MEC ≤0.015 mg/L), while olorofim activities varied by SCs, with MIC ≤0.25 mg/L against *Fusarium fujikuroi* SC. *FS*SC displayed higher voriconazole and amphotericin B MICs compared with other SCs, with *Neocosmospora keratoplastica* displaying a highest amphotericin B modal MIC of 4 mg/L. Four major *Neocosmospora* species showed voriconazole MIC ≥16 mg/L.

**Conclusions:**

Our findings indicated the two-step molecular algorithm accurately identifies *Fusarium* to species level. Further, we underscored the significance of considering both *Fusarium* SCs and species for predicting antifungal susceptibility, particularly to olorofim and amphotericin B.

## Introductions

*Fusarium* is listed as one of the WHO high priority fungal pathogens,^1^ reflecting its emergence within One Health frameworks and the current lack of effective antifungal agents. This genus is globally distributed across diverse environments and human habitats, serving as reservoirs to threaten health of human, animals and plants. *Fusarium* can be not only pathogens damaging crop widely and causing up to 50% yield loss in soybean, banana, tomato and wheat,^2^ but also cause human infections in the community and healthcare settings due to environment exposure. Most pathogenic species have been detected in environmental samples.^3^ Over 20 species complexes (SCs) exist in nature, with at least 7 SCs linked to human infections, ranging from superficial (such as keratitis and onychomycosis), locally invasive (cellulitis, sinusitis and pneumonia), to disseminated infections with positive blood cultures.^4,5^

Conventional antifungal agents offer limited efficacy against *Fusarium*; notably, *Fusarium solani* SC (*FS*SC) exhibit intrinsic resistance to azoles.^6,7^ Among different *Fusarium* SCs, susceptibilities to polyenes are variable. Among novel antifungal agents, manogepix targets Gwt1, a critical enzyme for synthesizing the glycosylphosphatidylinositol-anchored proteins of the fungal cell wall, and demonstrates notable *in vitro* activity against various *Fusarium* SCs.^8–10^ Olorofim, which inhibits dihydroorotate dehydrogenase, essential for the *de novo* pyrimidine synthesis pathway, may also exhibit *in vitro* activity against selected SCs.^11,12^ Yet, comparative data on the antifungal susceptibility profiles among species within the same SC remain scarce. In addition, routine implementation of antifungal susceptibility testing for *Fusarium* in clinical laboratories is challenged by technical limitations. There is ongoing interest in determining whether precise species identification can reliably predict *in vitro* susceptibility to novel and conventional antifungal agents.

Accurate species identification within a given *Fusarium* SC remains challenging due to morphological similarities that prevent accurate differentiation by conventional techniques. Furthermore, incomplete MALDI-ToF databases hinder identification capabilities in clinical laboratories.^13,14^ To overcome these limitations, molecular identification offers a robust alternative for species identification. While *ITS* is useful in the discrimination between the multiple SC of *Fusarium*, it lacks discriminatory power down to species level. Consequently, *TEF1α* is the preferred first-line marker for species identification. Also, it has higher PCR amplification and success rates compared with *RPB2.* Therefore, the current European Confederation of Medical Mycology and International Society for Human and Animal Mycology (ECMM/ISHAM) guideline recommends molecular identification using *ITS* and *TEF1*α for *Fusarium.*^15^ Although *RPB2* sequencing can resolve specific ambiguities of *TEF1α—*particularly within the *FS*SC and *F. fujikuroi* species complex (*FF*SC)—it remains uncertain whether additional taxonomic resolution correlates with a species-specific resistance pattern. We propose an efficient two-step molecular algorithm: initially sequence ITS and *TEF1*α (Step I), and, if species-level identification is inconclusive, sequence *RPB2* (Step II).

To address these gaps, we conducted a multicentre study to validate the two-step molecular algorithm for *Fusarium* species identification. We also compared distributions of *Fusarium* species identified by molecular methods from isolates causing fungaemia and cornea infections. In addition, we analysed *in vitro* susceptibilities of two antifungal agents under phase III clinical trials (manogepix, olorofim) and two currently preferred agents (amphotericin B, voriconazole) at both SC and individual species levels, aiming to determine the correlations between antifungal susceptibilities and *Fusarium* species.

## Methods

### Isolates

*Fusarium* clinical isolates were prospectively collected from patients at three hospitals between 2011 and 2023. The isolates were obtained from blood and cornea specimens, with two exceptions: one from an anterior chamber aspirate and one from contaminated contact lens solution. The following mycological studies were conducted at a reference laboratory at National Taiwan University Hospital (NTUH) after confirmation as *Fusarium* species and related genera by macroscopic colony morphology and microscopic features in a lactophenol wet mount preparation according to standard laboratory procedures. To account for potential epidemiological variations driven by geoclimatic differences, we stratified the study population into two geographically distinct groups: isolates from NTUH and NTUH Cancer centre located in subtropical Taiwan as a discovery cohort, and used isolates from National Cheng Kung University Hospital located in tropical Taiwan as a validation cohort.

### Sequencing for molecular identifications

*Fusarium* isolates were cultured on Sabouraud dextrose agar (Liofilchem, Via Scozia, Italy). Culture plates were incubated at 28°C and grown for up to 7 days. DNA extraction was performed by Quick-DNATM Fungal/Bacterial Miniprep Kit (Zymo Research, Irvine, USA). Three gene regions, *ITS*, *TEF1*α and *RPB2*, were amplified directly from the genomic DNA for sequencing using primer pairs ITS5/ITS4,^16^ EF1/EF2^17^ and RPB2–5f2, fRPB2–7cr, fRPB2–7cf and RPB2–11ar.^18,19^ The PCR conditions and primers were described and listed in the Supplementary Materials (available as Supplementary data at *JAC-AMR* Online) and Table S1, respectively.

All sequence results were uploaded and aligned to reference sequences deposited in the public databank (https://www.fusarium.org/) to confirm the species level of isolates tested. Based on the two-step algorithm, step I to achieve species identification was by using the nucleotide sequences of *ITS* and *TEF1α.* If both of *ITS* and *TEF1α* cannot determine a single species for a specific isolate, step II would use the full nucleotide sequence length of *RPB2* for species identification. If the alignment results in step II were unable to achieve single-species identification, the reporting rules were as follows: both species would be reported if only two species within the same species complex identified; otherwise, a species complex would be reported if ≥3 species within the species complex identified.

### Antifungal susceptibility testing

Broth microdilution methods for minimum effective concentrations (MEC) of manogepix (Cat no. HY-18233, MedChemExpress), and minimum inhibitory concentrations (MIC) of olorofim (Cat no. HY-104029, MedChemExpress), amphotericin B (Sigma-Aldrich) and voriconazole (Sigma-Aldrich) against all isolates were performed according to EUCAST E.Def 9.4 susceptibility testing with a final inoculum size of 1–2.5 × 10^5^ cfu/mL,^9,20^ except filtration (11-µm filter) of the inoculum was performed only if a significant number of hyphae are detected (>5% of fungal structures). The results were interpreted by EUCAST: tentative epidemiological cut-off values of amphotericin B for *FF*SC and *FS*SC were both ≤8 mg/L. *A. flavus* ATCC 20430404304 and *A. fumigatus* ATCC 20430504305 were used as controls.

### Statistics

MIC/MEC ranges, modal MIC and MIC_50_ and MIC_90_ (the MIC value that includes 50% and 90% of the isolates, respectively) values were calculated. Categorical variables were expressed by numbers (percentages) and compared by using the chi-square test with Bonferroni-adjusted α for pair-wise comparisons as *post hoc* analysis. A two-sided *P* value <0.05 was considered significant. All statistical analyses were performed using Stata software (version 17; StataCorp, College Station, TX, USA).

## Results

### Performance of the two-step molecular algorithm for *Fusarium*

**Figure 1.**
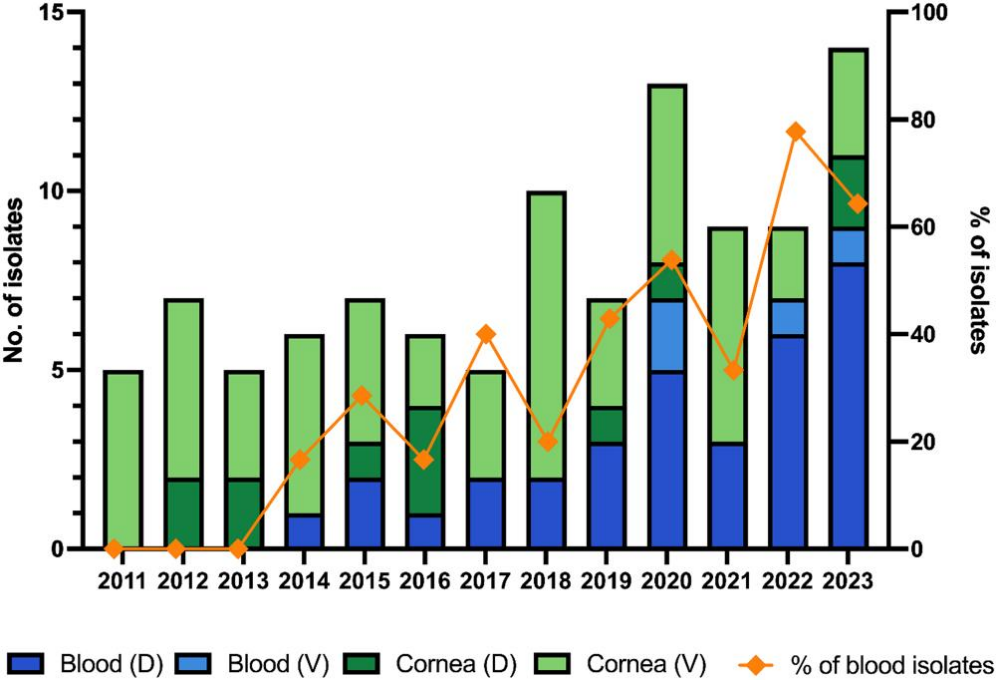
Distributions of *Fusarium* clinical isolates stratified by study cohort and specimens, and the trend of proportions of blood isolates during 2011 and 2023. D, discovery cohort; V, validation cohort.

**Figure 2.**
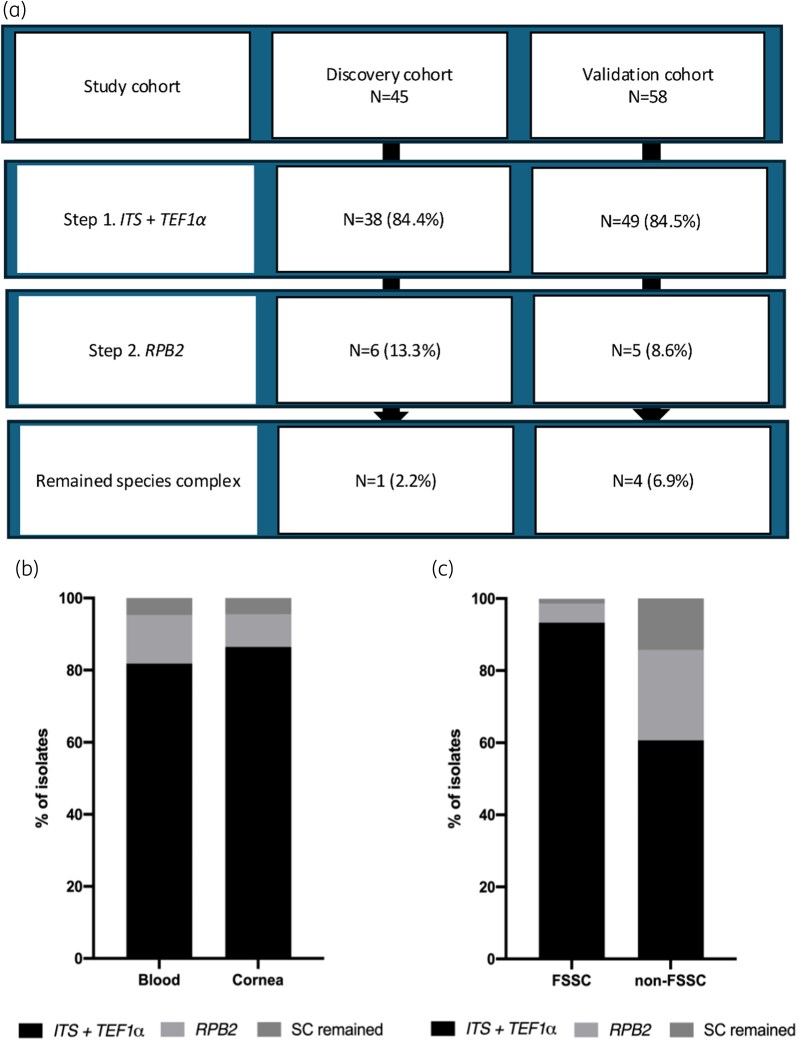
Distributions of *Fusarium* clinical isolates confirmed to species levels based on a two-step molecular identifications algorithm by study cohort (a), specimens (b) and SCs (c). *ITS*, internal transcribed spacer region of the nrDNA; *RPB2*, RNA polymerase second largest subunit; *TEF1α*, translation elongation factor 1 alpha.

Of 103 isolates collected from 96 patients evaluated, 37 (35.9%) were obtained from blood samples and 66 (64.1%) were isolated from cornea specimens. Notably, the proportions of blood isolates increased substantially over 13 years, from 0% to 64.3% (*P* for trend <0.001, Figure 1). In the discovery cohort (*n* = 45), initial step I identification classified 38 isolates (84.4%) to species levels, with step II increasing species-level identification by an additional 13.3% (*n* = 6, Figure 2a). Comparable results were observed in the validation cohort, with step I identifying most species level and an ≈9% increase after step II. After the two-step identification, 2.2% and 6.9% of isolates remained unidentified in the discovery and validation cohorts, respectively. The proportions of identification in each step were consistent between blood and cornea isolates (Figure 2b).

### Distributions of species complexes and species in *Fusarium*

*FS*SC was the predominant SC, accounting for 32 (78.4%) and 43 (65.2%) isolates in blood and cornea samples, respectively (Table 1). Overall, *FF*SC (*n* = 8, 7.8%) and *Fusarium dimerum* species complex (*FD*SC, *n* = 7, 6.8%) shared the second and third common species complex, followed by *Fusarium incarnatum–equiseti* species complex (*FIE*SC, *n* = 6), *Fusarium oxysporum* species complex (*FO*SC, *n* = 4) and other SCs (*n* = 3). After two steps of molecular identification all but one *FS*SC isolates were identified to species level, while 14.3% of non-*FS*SC isolates (4/28) remained classified only at SC level (*P* < 0.001) (Figure 2b). The proportion of *FS*SC in blood isolates were significantly greater than that of in cornea isolates [86.5% (32/37) versus 65.2% (43/66); *P* = 0.02].

**Table 1.** Distributions of *Fusarium* species by molecular identifications among blood and cornea specimens

Source	Species complex^a^	Species	Discovery cohort (*n* = 45)^b^	Validation cohort (*n* = 58)^b^	Total (*n* = 103)
Blood			33 (73.3)	4 (6.9)	37 (35.9)
	*FS*SC (*n* = 32)	*Neocosmospora bataticola*	1 (2.2)	0 (0)	1 (1.0)
		*Neocosmospora bostrycoides*	2 (4.4)	0 (0)	2 (1.9)
		*Neocosmospora diminuta*	1 (2.2)	0 (0)	1 (1.0)
		*Neocosmospora falciformis*	1 (2.2)	0 (0)	1 (1.0)
		*Neocosmospora ipomoeae*	1 (2.2)	0 (0)	1 (1.0)
		*Neocosmospora keratoplastica*	7 (15.6)	0 (0)	7 (6.8)
		*Neocosmospora petroliphila*	5 (11.1)	2 (3.4)	7 (6.8)
		*Neocosmospora pseudensiformis*	11 (24.4)	0 (0)	11 (10.7)
		*FS*SC (unknown species)	1 (2.2)	0 (0)	1 (1.0)
	*FF*SC (*n* = 2)	*Fusarium napiforme*	1 (2.2)	0 (0)	1 (1.0)
		*Fusarium planum*	1 (2.2)	0 (0)	1 (1.0)
	*FD*SC (*n* = 2)	*Bisifusarium dimerum*	1 (2.2)	1 (1.7)	2 (1.9)
	*FIE*SC (*n* = 1)	*FIE*SC (unknown species)^c^	0 (0)	1 (1.7)	1 (1.0)
Cornea			12 (26.7)	54 (93.1)	66 (64)
	*FS*SC (*n* = 43)	*Neocosmospora falciformis*	5 (11.1)	20 (34.5)	25 (24.3)
		*Neocosmospora ferruginea*	1 (2.2)	0 (0)	1 (1.0)
		*Neocosmospora keratoplastica*	3 (6.7)	10 (17.2)	13 (12.6)
		*Neocosmospora metavorans*	2 (4.4)	2 (3.4)	4 (3.9)
	*FF*SC (*n* = 6)	*Fusarium annulatum*	0 (0)	2 (3.4)	2 (1.9)
		*Fusarium erosum*	0 (0)	1 (1.7)	1 (1.0)
		*Fusarium proliferatum*	0 (0)	1 (1.7)	1 (1.0)
		*Fusarium pseudocircinatum*	0 (0)	2 (3.4)	2 (1.9)
	*FIE*SC (*n* = 6)	*Fusarium mucidum/Fusarium aberrans*	0 (0)	1 (1.7)	1 (1.0)
		*Fusarium tanahbumbuense*	1 (2.2)	1 (1.7)	2 (1.9)
		*FIE*SC (unknown species)^d^	0 (0)	3 (5.2)	3 (2.9)
	*FD*SC (*n* = 5)	*Bisifusarium delphinoides*	0 (0)	2 (3.4)	2 (1.9)
		*Bisifusarium lovelliae*	0 (0)	2 (3.4)	2 (1.9)
		*Bisifusarium nectrioides*	0 (0)	1 (1.7)	1 (1.0)
	*FO*SC (*n* = 4)	*Fusarium cugenangense*	0 (0)	2 (3.4)	2 (1.9)
		*Fusarium liriopes*	0 (0)	1 (1.7)	1 (1.0)
		*Fusarium nirenbergiae*	0 (0)	1 (1.7)	1 (1.0)
	*FDEC*SC (*n* = 1)	*Albonectria rigidiuscula*	0 (0)	1 (1.7)	1 (1.0)
	*FN*SC (*n* = 1)	*Fusarium commune*	0 (0)	1 (1.7)	1 (1.0)

*FDEC*SC, *Fusarium decemcellulare* species complex; *FN*SC, *Fusarium nisikadoi* species complex.

^a^ *P* for SCs distributions between discovery and validation cohort, 0.01; for those between blood and cornea, 0.25.

^b^Isolate numbers and percentages in bold indicate the top species of blood and cornea samples in the two cohorts, respectively.

^c^Could be *Fusarium sulawesiense*, *F. pernambucanum*, *F. caatingaense*, *F. irregulare*, *F. multiceps*, *F. luffae*, *F. annulatum* or *F. persicinum*.

^d^NCK0034 could be *Fusarium sulawesiense*, *F. pernambucanum* or *F. caatingaense.* NCK1044 and NCK1070 could be *Fusarium sulawesiense*, *F. pernambucanum*, *F. caatingaense*, *F. irregulare*, *F. multiceps*, *F. luffae*, *F. annulatum* or *F. persicinum*.

Within *FS*SC, blood isolates demonstrated the relatively even distribution among *Neocosmospora pseudensiformis* (34.3%, 11/32), *Neocosmospora keratoplastica* and *Neocosmospora petroliphila* (both of each, 21.9% [7/32]), while cornea isolates showed *Neocosmospora falciformis* (58.1%, 25/43) as the predominant species, followed by *N. keratoplastica* (30.2%, 13/43). By contrast, non-*FS*SC species in cornea samples exhibited extensive diversity: *FF*SC belonged to four species (*n* = 6), *FD*SC belonged to three species (*n* = 5), *FIESC* belonged to at least two species (*n* = 6) and *FO*SC belonged to three species (*n* = 4).

### *In vitro* susceptibilities of antifungal agents against *Fusarium*

The distribution of amphotericin B MICs ranged widely (0.125–>16 mg/L) and varied by SCs with variable modal MICs (0.5–2 mg/L) in four major SCs and highest MIC_90_ for *FIE*SC (>16 mg/L) (Table 2). *In vitro* activities of voriconazole were very poor against all SCs, with modal MICs ≥4 mg/L and all MIC_90_ > 16 mg/L. Manogepix exhibited potent and consistent activity across all 103 isolates, with MEC values at the lowest tested concentrations of ≤0.015 mg/L in each of SCs. Olorofim MICs also ranged widely and varied by SCs. Olorofim was highly active against *FF*SC with a model MIC of 0.008 mg/L, while its MIC_50_/MIC_90_ values for *FS*SC, *FD*SC and *FIE*SC were higher than the upper limit of the tested concentrations, 0.5 mg/L.

**Table 2.** Antifungal minimum inhibitory/effective concentrations against Fusarium species by SCs

	Amphotericin B MIC (mg/L)			Voriconazole MIC (mg/L)			Manogepix MEC (mg/L)			Olorofim MIC (mg/L)		
	Modal^a^	MIC_50_/MIC_90_^a^	Range	Modal^a^	MIC_50_/MIC_90_^a^	Range	Modal^a^	MEC_50_/MEC_90_^a^	Range	Modal^a^	MIC_50_/MIC_90_^a^	Range
*FS*SC (*n* = 75)	1	1/4	0.125–>16	>16	>16/>16	0.06–>16	≤0.015	≤0.015	≤0.015	>0.5	>0.5/>0.5	>0.5
*FF*SC (*n* = 8)	2	2/4	0.5–4	4	4/>16	1–>16	≤0.015	≤0.015	≤0.015	0.008	0.008/0.25	0.008–0.25
*FD*SC (*n* = 7)	0.5	0.5/2	0.25–2	16	4/>16	2–>16	≤0.015	≤0.015	≤0.015	>0.5	>0.5/>0.5	>0.5
*FIE*SC (*n* = 6)	1	2/>16	1–>16	8	8/>16	0.125–>16	≤0.015	≤0.015	≤0.015	>0.5	>0.5/>0.5	>0.5
*FO*SC (*n* = 4)	NA^a^	NA^a^	1–8	NA^a^	NA^a^	8–>16	NA^a^	NA^a^	≤0.015	NA^a^	NA^a^	0.25–>0.5
Others (*n* = 3)^8^	NA^a^	NA^a^	0.5–>16	NA^a^	NA^a^	1–>16	NA^a^	NA^a^	≤0.015	NA^a^	NA^a^	>0.5

MIC_50_ and MIC_90_ represent the lowest concentration of the antifungal at which 50% and 90% of the isolates were inhibited, respectively; NA, not applicable.

^a^Modal MIC/MEC, MIC_50_/MEC_50_ and MIC_90_/MEC_90_ were calculated for SCs with ≥5 isolates.

^b^The other group included each of the isolates belonging to the *Fusarium decemcellulare* species complex, *F. nisikadoi* species complex and *Fusarium* species.

Overall, *FS*SC generally displayed higher MICs for antifungal agents tested except manogepix. Of note, the ranges of amphotericin B and voriconazole MICs against *FSSC* distributed widely (0.125–>16 mg/L, and 0.06–>16 mg/L, respectively). Thus, we further analysed antifungal susceptibilities of these two preferred available antifungal agents to species level within *FS*SC (*n* ≥ 5 per species). *N. keratoplastica*, the second most common species, had the highest modal MIC for amphotericin B at 4 mg/L, while those of *N. falciformis*, *N. pseudensiformis* and *N. petroliphil* were 0.5–1 mg/L (Table 3). Nevertheless, the proportion of non-wild-type isolates to amphotericin B was greatest in *N. pseudensiformis* (18.2%), compared with *N. keratoplastica* (5.0%), *N. falciformis* (3.8%) and *N. petroliphila* (0%), resulting in similar geometric means (GMs) MICs for *N. pseudensiformis* and *N. keratoplastica* (3.31 versus 3.03 mg/L). For voriconazole, most isolates in four common *Neocosmospora* species showed MICs ≥16 mg/L.

**Table 3.** Distributions of antifungal MIC against common *Neocosmospora* (*n*  ≥  5) by species

	MICs (mg/L)^a^											
	≤0.03	0.06	0.12	0.25	0.5	1	2	4	8	≥16	GM	Non-WT, *n* (%)^b,c^
*N. falciformis* (*n* = 26)												
Amphotericin B	0	0	0	0	7	**12**	5	1	0	1	1.14	1 (3.8)
Voriconazole	0	0	0	0	0	0	0	1	0	**25**	≥16	NA
*N. keratoplastica* (*n* = 20)												
Amphotericin B	0	0	0	0	0	2	7	**9**	1	1	3.03	1 (5.0)
Voriconazole	0	0	0	0	0	0	0	0	0	**20**	≥16	NA
*N. pseudensiformis* (*n* = 11)												
Amphotericin B	0	0	1	0	0	**5**	2	1	0	2	3.31	2 (18.2)
Voriconazole	0	0	0	0	0	0	0	0	0	**11**	≥16	NA
*N. petroliphila* (*n* = 8)												
Amphotericin B	0	0	0	0	**4**	1	1	2	0	0	1.22	0 (0)
Voriconazole	0	0	0	0	0	0	0	0	0	**8**	≥16	NA

GM, geometric means; NA, not applicable.

^a^Isolate numbers in bold indicate modal MICs.

^b^The minimum inhibitory concentration results were interpreted by EUCAST: tentative epidemiological cut-off values of amphotericin B for *FF*SC and *FS*SC were both ≤8 mg/L.

^c^ *P* for overall comparisons of non-WT type proportions, 0.31.

## Discussions

This current study is the largest series of *Fusarium* clinical isolates incorporating those obtained from blood cultures (Table S2) and demonstrates a two-step molecular identification protocol facilitating precise species-level classification in >90% of isolates evaluated. We identified *FS*SC to be the major pathogenic SC overall, and more commonly found in blood than cornea samples among seven SCs. Besides, the species distributions of *FS*SC varied by clinical samples. Of four antifungal agents evaluated, manogepix exhibited potent activity against all isolates, while olorofim activities varied by SCs. MICs of amphotericin B and voriconazole varied at SC and species levels. Overall, *FS*SC generally exhibited higher MICs for amphotericin B and voriconazole compared with non-*FS*SC. All these findings not only reinforced the robustness of current international guidelines for laboratory identification of this clinically relevant genus, but also elucidates the correlations between antifungal susceptibilities and *Fusarium* species, particularly for *FS*SC.

The molecular identification of *Fusarium* by *ITS* and the *TEF1α* gene is currently recommended by international medical guidelines and agriculture societies, while *RPB2* allows for enhanced discrimination between closely related species of *FFSC* and *FSSC.*^15,21,22^ However, previous clinical studies focused on analyses of molecular identification of *Fusarium* by the *TEF1α* gene only (Table S2).^13,14,23–25^ This study is among the first, to best of our knowledge, of the clinical studies to test the molecular identification of *Fusarium* from clinical samples by two steps encompassing *ITS*, *TEF1α* and *RPB2*. The decision to incorporate *RPB2* sequencing was driven by its ability to resolve the closely related species within *FSSC* and *FFSC* that remain undistinguished by *TEF1α* alone. Uniquely, this study quantified the clinical yield of this approach: while *ITS* and *TEF1α* correctly identified >80% of clinical isolates, the second step of *RPB2* sequencing successfully identified a further 10% (11/103). Notably, this added resolution was not limited to *F*SSC (*n* = 4), but also identified species within *FIE*SC (*n* = 3), *FD*SC (*n* = 2), *FF*SC (*n* = 1) and *FO*SC (*n* = 1) (Figure 2c). Our results were consistent in both discovery and validation cohorts, supporting the recommendations by current guidelines and consensus to use *RPB2* for resolving ambiguities in *TEF1α* analysis.^15,21,22^

In this study, most clinical *Fusarium* isolates belonged to *FSSC*, aligning with previous reports from tropical or subtropical countries, including two studies conducted in Taiwan.^14,23–28^ The prevalence of specific *Fusarium* SCs varied by epidemiological factors. First, geographic variations matter (Table S2). Specifically, *FS*SC is dominant in Asia and South America, in addition to latitudinal differences.^14,23–29^ In Europe and the USA, two different multicentre studies found FOSC is the leading SC (35.7% and 73.8%, respectively).^11,13^ Second, the sample sources differ, resulting in variations of the distributions of *Fusarium* SCs even under the similar climatic condition. For instance, a multicentre study in France reported the most common SC isolated from blood as over a 10-year period was *FF*SC (53.7%, 29/54), rather than *FO*SC.^30^ In addition, our study found the proportions of *FS*SC differed between blood and cornea samples. Third, patient populations also differ. A multicentre study from Spain between 2000 and 2015 indicated non-neutropenic patients often have localized fusariosis with dominant unknown *Fusarium* SCs (63.2%), while neutropenic patient had more frequently have disseminated fusariosis with *FS*SC as the top SC (38.5%).^31^ These observations highlight the need for ongoing monitoring of clinical *Fusarium* epidemiology, considering patient groups, sample types and regional differences.

On the other hand, ocular trauma represents the most common prevalent cause of *Fusarium* keratitis in settings without contact lens-related outbreaks, with patients often exposed to *Fusarium* via contaminated soil or plants in nature.^24,26,27^ In this study, a total of 23 isolates obtained from cornea samples were clustered to 14 species within six non-*FS*SC, highlighting the extensive diversity of *Fusarium* in the environment. Of note, we identified the first human keratitis caused by *Albonectria rigidiuscula* at a hospital located in tropical Taiwan. This species, belonging to *Fusarium decemcellulare* species complex, is recognized as an important phytopathogen affecting common agriculture plants, such as mango, in tropical areas including Taiwan.^21,22^ Given *Fusarium* consists of at least 300 phylogenetically distinct species and 23 SCs,^21,22^ our result underscored that *Fusarium* is a continuous threat to human health from a One Health perspective.

As for *in vitro* susceptibility testing, manogepix exhibited consistent activity against a range of *Fusarium* SCs in this study, aligning with previous findings obtained using either the EUCAST or CLSI methodologies.^8–10,32^ Although earlier studies were conducted primarily in the USA and Europe, the Asia–Pacific area remains critical for *Fusarium* infections, as shown in Table S2. This report systemically investigates by manogepix MECs using the EUCAST method, providing comprehensive and timely data that show manogepix MECs at the lowest tested concentrations across all *Fusarium* isolates. These results support the implementation of the ongoing phase III clinical trial of fosmanogepix, a prodrug of manogepix, for treatment of adult patient with invasive fusariosis, especially those with high MIC of amphotericin B and voriconazole for *FS*SC, as well as other mould infections.^33^

We found olorofim, another novel antifungal agent, showed limited *in vitro* activities against variable *Fusarium* SCs, except *FF*SC by measured at 90% inhibition of growth. Previous studies by the same reading endpoints have shown consistent high olorofim MICs against *FS*SC, *FD*SC and *FIE*SC.^11,12,32^ While these studies found differences in susceptibility at species level within *FF*SC and *FO*SC, our results showed uniformly low olorofim MICs against *FF*SC and high MICs against *FO*SC. In addition to relatively small numbers of *FF*SC (*n* = 8) and *FO*SC (*n* = 4) in our study, species difference with a specific SC may contribute to the discordant results. For example, one study by the EUCAST method found olorofim GMs of MICs for *F. jonfreemaniae* and *F. musae* was greater than that of *F. annulatum* and *F. verticillioides* (0.445 versus 0.124 versus 0.018 versus 0.081 mg/L) within 41 *FF*SC isolates.^32^ In our cohort, among eight *FF*SC isolates, only one *F. annulatum* was identified and showed a comparable olorofim MIC of 0.015 mg/L, while the other species differed from those in that study. These findings suggested that species-level identification within *Fusarium* SC is crucial for predicting antifungal susceptibility.

Regarding amphotericin B and voriconazole, two preferred agents for initial therapy of invasive fusariosis, this study found that *FS*SC displays higher MICs compared with non-*FSSC*, which is consistent with previous studies.^7,14,24,34^ Overall, *in vitro* activities of voriconazole were very poor against all SCs, with modal MICs ≥4 mg/L and all MIC_90_ > 16 mg/L. Furthermore, the distribution of amphotericin B MICs ranged from 0.125 µg/mL to more than 16 µg/mL for *FS*SC. By applying two-step molecular identification, we were able to demonstrate this variation is not random, but rather species-dependent within *FS*SC. Of note, *N. keratoplastica*, the second most common species of *FS*SC, exhibited higher amphotericin modal MICs (4 µg/mL) compared with other *Neocosmospora* species (0.5–1 mg/L), consistent to previous reports.^14^ While the proportion of non-wild type (WT) to amphotericin B (16 µg/mL or more) was highest for *N. pseudensiformis* (18%), the most common species of *FS*SC. Because commercial antifungal susceptibility tests for *Fusarium* have not yet been verified, molecular identification of *Fusarium*, especially *FS*SC, in conjunction with local susceptibility data may assist clinicians in selecting appropriate antifungal agents and/or titrate the optimal dose of amphotericin B.

The current study has several limitations. First, 70.8% of our clinical isolates were *FS*SC. Therefore, caution is advised when applying the two-step molecular identification and interpreting antifungal susceptibility results for non-*FS*SC or non-clinical isolates. In Netherland, a study focusing on *FF*SC also demonstrated antifungal resistance patterns are species specific.^35^ Hence, species-level identification among non-*FS*SC may be practicable for the choice of antifungal treatment as well. Second, *in vitro* susceptibility testing for both novel and conventional antifungal agents were primarily conducted on *FS*SC with variable species. For accurate comparison with other studies, species-level identification within *Fusarium* SC is necessary, as species variations in susceptibility to a specific antifungal agent were observed. Third, regarding the clinical feasibility, we acknowledge that routine implementation of this two-step molecular strategy may be restricted by costs and technical capabilities. Therefore, we propose integrating this method into a tiered workflow, where molecular sequencing is reserved for isolates that fail species-level identification by MALDI-TOF MS. However, implementation of even this targeted approach remains challenging, as a recent survey indicated that DNA sequencing for fungal identification is available in only one-third of clinical laboratories in the Asia–Pacific region.^36^ Hence, future multicentre studies are needed to validate these findings and, crucially, to use this molecularly characterized dataset to expand and refine MALDI-TOF MS reference databases, bridging the gap between high-resolution sequencing and rapid routine diagnostics. Fourth, our study lacked clinical data, which limits the ability to correlate antifungal MICs and clinical outcomes. Yet, previous research indicated that a clear correlation between *in vitro* activity and clinical effectiveness in invasive fusariosis may not exist.^7^

Collectively, the multicentre study demonstrates the utility of a two-step molecular identification protocol for accurately classifying *Fusarium* clinical isolates at the species level. These results refine understanding of the local epidemiology of blood and cornea *Fusarium* species, highlighting the predominance of *FS*SC and potential geographic variations at both SC and individual species levels. Manogepix displayed outstanding *in vitro* activities against all *Fusarium* isolates at the lowest tested concentrations. In comparison, susceptibilities to olorofim and amphotericin B had variations by SCs and/or species. These findings illustrate the significance of considering both species and species complex when predicting antifungal susceptibility, and such an alternative method may facilitate streamlined selecting optimal antifungal agents in clinical settings.

## Supplementary Material

dlag022_Supplementary_Data

## Data Availability

The DNA sequences of all isolates in this study were deposited in GenBank (http://www.ncbi.nlm.nih.gov/) under BioProject accession number: PRJDB37776.
