# Supplementary material for: Assessment of the molecular identification algorithm and its impact on antifungal susceptibilities against clinical Fusarium isolates: a multicentre study in Taiwan, 2011–2023
Source: JAC Antimicrob Resist. 2026 Feb 26;8(1):dlag022. doi: 10.1093/jacamr/dlag022 (PMC12936585; doi:10.1093/jacamr/dlag022)
Supplement: dlag022_Supplementary_Data [file dlag022_supplementary_data.docx]

**Supplementary Appendix to** Assessment of the molecular identification algorithm and its impact on antifungal susceptibilities against clinical *Fusarium* isolates: A multicentre study in Taiwan, 2011–2023.

Pao-Yu CHEN^1^, Chi-Jung WU^2,3^, Un-In WU^1^, Wang-Da LIU^4^, Yee-Chun CHEN^1,3,4*^

^1^ Division of Infectious Diseases, Department of Internal Medicine, National Taiwan University Hospital, Taipei, Taiwan

^2^ Division of Infectious Diseases, Department of Internal Medicine, National Cheng Kung University Hospital, College of Medicine, National Cheng Kung University, Tainan, Taiwan

^3^ National Institute of Infectious Diseases and Vaccinology, National Health Research Institutes, Miaoli, Taiwan

^4^ Department of Medicine, National Taiwan University Cancer Center, Taipei, Taiwan

^5^ School of medicine, National Taiwan University College of Medicine, Taipei, Taiwan

**Table of Contents**

Supplementary methods……………………………………………………………………………………….….2

Supplementary Table S1……………………………………………………………………………………………3

Supplementary Table S2……………………………………………………………………………………………4

Supplementary reference………………………………………………………………………………………….6

**Supplementary materials**

The PCR conditions were described as follows: PCR reaction mixture (25 μL final volume) contained 10 × PCR buffer 2.5 μL, water 16.375 μL, dNTP mix (2.5 mm) 2 μL, 1 μL of each primer (10 pmol), Taq polymerase (5 U/μL) 0.125 μL, and template DNA (100 ng/μL) 2 μL. Amplification was performed in a Biometra T3000 (Biometra GmbH, Konrad-Zuse-Strasse 1, Germany) thermocycler as follows: 95°C for 5 min, followed by 35°Cycles consisting of 95°C for 30 sec, 55°C for 30 sec and 72°C for 45 sec, and a delay at 72°C for 10 min. Annealing temperature was changed to 60°C for the *TEF1*α gene. Thermocycle for the *RPB2* gene was as follow: 95°C for 5 min, followed by 35°Cycles consisting of 95°C for 1 min, 60°C for 2 min and 72°C for 2 min, and a delay at 72°C for 10 min. PCR products were visualized by electrophoresis on a 1.2% (w/v) agarose gel. Sequencing was done on an ABI 3730xL automatic sequencer (Applied Biosystems, Waltham, USA).

**Supplementary Table 1. PCR primers for DNA amplification of *Fusarium* and related genera.**

| **Gene/DNA region Primer** | |  | | | **Name** | | | **Direction** | | | **Sequence (5'→3')** | | **Reference** | |
| --- | --- | --- | --- | --- | --- | --- | --- | --- | --- | --- | --- | --- | --- | --- |
| **Name** | **Abbreviation** |  |  | | | |  | | |  | |  | | |
| Internal transcribed spacer region of the nrDNA | *ITS* | | |  | | ITS5 | | | Forward | | GGAAGTAAAAGTCGTAACAAGG | | | [1] |
|  |  | | |  | | ITS4 | | | Reverse | | TCCTCCGCTTATTGATATGC | | | [1] |
| Translation elongation factor 1-alpha | *TEF1α* | | |  | | EF-1 | | | Forward | | ATGGGTAAGGARGACAAGAC | | | [2] |
|  |  | | |  | | EF-2 | | | Reverse | | GGARGTACCAGTSATCATG | | | [2] |
| RNA polymerase second largest subunit | *RPB2* | | |  | | RPB2-5f2 | | | Forward | | GGGGWGAYCAGAAGAAGGC | | | [3] |
|  |  | | |  | | fRPB2-7cf | | | Forward | | ATGGGYAARCAAGCYATGGG | | | [4] |
|  |  | | |  | | fRPB2-7cr | | | Reverse | | CCCATRGCTTGYTTRCCCAT | | | [4] |
|  |  | | |  | | RPB2-11ar | | | Reverse | | GCRTGGATCTTRTCRTCSACC | | | [4] |

**Abbreviations:** *ITS,* Internal transcribed spacers of ribosomal DNA*; TEF1*α, translation elongation factor 1-alpha; *RPB2*, the second largest RNA polymerase subunit.

**Supplementary Table 2. Comparisons of numbers, primers, species complex distributions, and specimen sources of clinical isolates belonged to *Fusarium* and related genera by molecular identifications to species levels in the literature.**

| **Study**^a^ | **Country/**  **region** | **Study period** | **Genes for molecular id** | **Numbers of clinical isolates^b^** | | | | | | | **Source^b^** | **REF** |
| --- | --- | --- | --- | --- | --- | --- | --- | --- | --- | --- | --- | --- |
|  |  |  |  | **Total** | ***FS*SC** | ***FF*SC** | ***FO*SC** | ***FD*SC** | ***FIE*SC** | **Other SCs** |  |  |
| Muraosa *et al*. (2017) | Japan^a^ | 1998–2015 | *ITS, TEF1 α, RPB2* | 73 | **53** | 7 | 8 | 3 | 2 | 0 | **Eye, 36**; blood, 15; skin, 11; nail, 5; urine, 3; sinus, 1; ileocecal tissue, 1. | [5] |
| Herkert *et a*l. (2019) | Brazil^a^ | 1985–2015 | *TEF1 α* | 43 | **40** | 3 | 0 | 0 | 0 | 0 | **Skin, 24**; blood, 10; eye, 5; synovial fluid, 3; airway, 1. | [6] |
| Bansal *et al*. (2019) | India | 2012-2014 | *ITS, NL4* | 33 | **13** | 13 | 0 | 0 | 7 | 0 | **Eye, 16**; nail, 14; airway, 2; sinus, 1 | [7] |
| Normand *et al.* (2021) | Europe^a^ | 2018 | *TEF1 α* | 160 | 50 | 48 | **54** | 4 | 4 | 2^d^ | **Colonization, 62**; nails, 50; eye, 37; invasive isolates, 11. | [8] |
| Guo *et al*. (2022) | China^a^ | 2018–2020 | *TEF1 α* | 95 | **67** | 16 | 7 | 2 | 1 | 2^e^ | **Eye, 45**; skin, 38; pus, 4; blood, 4; airway, 3; urine, 1. | [9] |
| Huang *et a*l. (2022) | Taiwan | 2015–2020 | *ITS, TEF1 α* | 43 | **33** | 2 | 2 | 4 | 2 | 0 | **Eye, 43.** | [10] |
| James *et al*. (2022) | Malaysia^a^ | 2007-2011 | *TEF1 α, RPB2* | 15^c^ | **15** | 0 | 0 | 0 | 0 | 0 | **Nail, 8**; eye, 3; skin, 2; blood, 2. | [11] |
| Ferreira da Cunha Neto *et al*. (2024) | Brazil | 2012–2022 | *TEF1 α, RPB2* | 60 | **47** | 9 | 4 | 0 | 0 | 0 | **Eye, 50**; nail & skin, 10. | [12] |
| Milanez *et al*. (2024) | Brazil | 2019–2022 | *TEF1 α, RPB2* | 27 | **14** | 6 | 0 | 7 | 0 | 0 | **Eye, 42^h^.** | [13] |
| Chen *et al.* (2025) | Taiwan^a^ | 2001–2022 | *ITS, TEF1 α, RPB1* | 41 | **28** | 3 | 2 | 3 | 5 | 0 | **Blood, 15**; eye, 13; airway, 6; skin, 5; nail, 1; ascites, 1. | [14] |
| Garbe *et al*. (2025) | Germany^a^ |  | *TEF1 α, RPB2* | 142 | **45** | 41 | 35 | 12 | 5 | 4^f^ | **Eye, 68**; skin & wound, 28; airway, 23; blood, 13; others, 13. | [15] |
| Roman-Montes *et al*. (2025) | Mexico | 2014–2021 | *TEF1 α* | 35 | **18** | 6 | 7 | 3 | 1 | 0 | **Airway, 11**; blood, 1; GI tract, 1^i^. | [16] |
| Chen *et al*. (2025) | Taiwan^a^ | 2011–2023 | Step I: *ITS, TEF1 α,* Step II: *RPB2* | 103 | **75** | 8 | 4 | 7 | 7 | 2^g^ | **Eye, 66**; blood, 37. | Current study |

**Abbreviations:** *FDSC*, *Fusarium dimerum* species complex; *FFSC*, *Fusarium fujikuroi* species complex; *FIESC*, *Fusarium incarnatum-equiseti* species complex, including *Fusarium camptoceras* species complex; *FOSC*, *Fusarium oxysporum* species complex; *FSSC,* *Fusarium solani* species complex; NR, not reported.

^a^ These studies were conducted as a multicenter design.

^b^ Isolate numbers in **bold** indicate the most common SC or source, and those with underline indicate blood isolates.

^c^ This study only included *FS*SC.

^d^ One of each isolate belonged to *Fusarium sambucinum* species complex and *Fusarium redolens* species complex, respectively.

^e^ One of each isolate belonged to *Fusarium chlamydosporum* species complex and *Fusarium nisikadoi* species complex, respectively.

^f^ Four isolate all belonged to *Fusarium redolens* species complex

^g^ One of each isolate belonged to *Fusarium decemcellulare* species complex and *Fusarium nisikadoi* species complex, respectively.

^h^ Only 27 isolates were randomly selected for molecular identification.

^i^ Only 13 isolates were reported specimen sources.

**Supplementary reference**

1. White TJ, Bruns TD, Lee SB, Taylor JW. Amplification and Direct Sequencing of Fungal Ribosomal RNA Genes for Phylogeneti*.* Academic Press, Inc.; 1990.

2. O'Donnell K, Kistler HC, Cigelnik E, Ploetz RC. Multiple evolutionary origins of the fungus causing Panama disease of banana: concordant evidence from nuclear and mitochondrial gene genealogies. Proc Natl Acad Sci U S A. 1998;**95**:2044–9.

3. Reeb V, Lutzoni F, Roux C. Contribution of RPB2 to multilocus phylogenetic studies of the euascomycetes (Pezizomycotina, Fungi) with special emphasis on the lichen-forming Acarosporaceae and evolution of polyspory. Mol Phylogenet Evol. 2004;**32**:1036–60.

4. Liu YJ, Whelen S, Hall BD. Phylogenetic relationships among ascomycetes: evidence from an RNA polymerse II subunit. Mol Biol Evol. 1999;**16**:1799–808.

5. Muraosa Y, Oguchi M, Yahiro M, Watanabe A, Yaguchi T, Kamei K. Epidemiological Study of *Fusarium* Species Causing Invasive and Superficial Fusariosis in Japan. Med Mycol J. 2017;**58**:E5–E13.

6. Herkert PF, Al-Hatmi AMS, de Oliveira Salvador GL, et al. Molecular Characterization and Antifungal Susceptibility of Clinical *Fusarium* Species From Brazil. Front Microbiol. 2019;**10**:737.

7. Bansal Y, Singla N, Kaistha N, Sood S, Chander J. Molecular identification of *Fusarium* species complex isolated from clinical samples and its antifungal susceptibility patterns. Curr Med Mycol. 2019;**5**:43–9.

8. Normand AC, Imbert S, Brun S, et al. Clinical Origin and Species Distribution of *Fusarium* spp. Isolates Identified by Molecular Sequencing and Mass Spectrometry: A European Multicenter Hospital Prospective Study. J Fungi (Basel). 2021;**7**:246.

9. Guo P, Chen J, Tan Y, et al. Comparison of molecular and MALDI-TOF MS identification and antifungal susceptibility of clinical *Fusarium* isolates in Southern China. Front Microbiol. 2022;**13**:992582.

10. Huang TE, Ou JH, Hung N, et al. *Fusarium* Keratitis in Taiwan: Molecular Identification, Antifungal Susceptibilities, and Clinical Features. J Fungi (Basel). 2022;**8**:476.

11. James JE, Santhanam J, Zakaria L, et al. Morphology, Phenotype, and Molecular Identification of Clinical and Environmental *Fusarium solani* Species Complex Isolates from Malaysia. J Fungi (Basel). 2022;**8**:845.

12. Ferreira da Cunha Neto J, da Silva Rocha WP, Makris G, et al. Fusarioid keratitis and other superficial infections: A 10-years prospective study from Northeastern Brazil. PLoS Negl Trop Dis. 2024;**18**:e0012247.

13. Milanez EPR, de Souza P, Monteiro RC, et al. *Fusarium* keratitis in a Brazilian tropical semi-arid area: Clinical-epidemiological features, molecular identification and antifungal susceptibility. Mycoses. 2024;**67**:e13728.

14. Chen YC, Ou JH, Wu CJ, et al. Clinical and Hospital Environmental *Fusarium* in Taiwan: Molecular Identification, Antifungal Susceptibilities, and Phylogenetic Analyses. Mycoses. 2025;**68**:e70056.

15. Garbe E, Ullah A, Aldejohann AM, Kurzai O, Janevska S, Walther G. *In vitro* activity of novel antifungals, natamycin, and terbinafine against *Fusarium*. Antimicrob Agents Chemother. 2025;**69**:e0191324.

16. Roman-Montes CM, Gonzalez-Lara F, Diaz-Lomeli P, et al. Molecular Identification and Antifungal Susceptibility of *Fusarium* spp. Clinical Isolates. Mycoses. 2025;**68**:e70012.
